# Identification of p72 epitopes of African swine fever virus and preliminary application

**DOI:** 10.3389/fmicb.2023.1126794

**Published:** 2023-02-03

**Authors:** Chun Miao, Sicheng Yang, Junjun Shao, Guangqing Zhou, Yunyun Ma, Shenghui Wen, Zhuo Hou, Decai Peng, HuiChen Guo, Wei Liu, Huiyun Chang

**Affiliations:** ^1^African Swine Fever Regional Laboratory of China (Lanzhou), State Key Laboratory of Veterinary Etiological Biology, Lanzhou Veterinary Research Institute, Chinese Academy of Agricultural Sciences, Lanzhou, Gansu, China; ^2^Animal Science and Technology College, Guangxi University, Nanning, Guangxi, China

**Keywords:** African swine fever virus, p72, monoclonal antibodies, epitope, diagnosis

## Abstract

African swine fever virus (ASFV) causes a highly lethal hemorrhagic viral disease (ASF) of pigs that results in serious losses in China and elsewhere. The development of a vaccine and diagnosis technology for ASFV is essential to prevent and control the spread of ASF. The p72 protein of ASFV is highly immunogenic and reactive, and is a dominant antigen in ASF vaccine and diagnostic research. In this study, 17 p72 monoclonal antibodies (mAbs) were generated. Epitope mapping by a series of overlapping peptides expressed in *Escherichia coli* showed that these mAbs recognized a total of seven (1–7) linear B cell epitopes. These mAbs did not show significant neutralizing activity. Epitopes 1 (^249^HKPHQSKPIL^258^), 2 (^69^PVGFEYENKV^77^), 5 (^195^VNGNSLDEYSS^205^), and 7 (^223^GYKHLVGQEV^233^) are novel. Sequence alignment analysis revealed that the identified epitopes were highly conserved among 27 ASFV strains from nine genotypes. Preliminary screening using known positive and negative sera indicated the diagnostic potential of mAb-2B8D7. The results provide new insights into the antigenic regions of ASFV p72 and will inform the diagnosis of ASFV.

## 1. Introduction

African swine fever (ASF) is an acute, contagious, and pathogenic infectious disease caused by African swine fever virus (ASFV). The virus is the only member of the *Asfarviridae* and the only known DNA arbovirus (Gaudreault et al., 2020; Ruiz-Saenz et al., 2022). The ASFV linear, double-strand DNA genome is 170–190 kbp in length (Dixon et al., 2013). It encodes 68 structural proteins and 150–200 non-structural proteins (Wang Y. et al., 2021). Recent studies have resolved the large icosahedral structure of ASFV (Wang et al., 2019; Li et al., 2020). From outside-in, the virus particles are composed of an outer envelope, capsid, inner membrane, core shell, and the genome (Wang et al., 2019; Wang Y. et al., 2021).

ASF cases were first reported in Kenya in 1921, with subsequent spread to western Europe and Latin America (Gaudreault et al., 2020). In 2007, ASF was reported in Georgia in the Caucasus and then spread to the northern and eastern Europe, with huge economic losses (Vergne et al., 2016; Jia et al., 2017; Iscaro et al., 2022). Since outbreaks in China in 2018, the dispersion of ASF accelerated to include Thailand, Laos, and Cambodia (Cadenas-Fernández et al., 2022). ASF was reintroduced in the Dominican Republic and Haiti after July 2021 (Gonzales et al., 2021; Urbano and Ferreira, 2022). ASF has caused huge losses to the global pig and related industries. There are no commercial ASF vaccines available (Cadenas-Fernández et al., 2022; Franzoni et al., 2022). The development of safe and effective vaccines is crucial. Current research on ASFV vaccines focuses on inactivated (Blome et al., 2020), attenuated (Borca et al., 2020; Gladue et al., 2021), and subunit vaccines (Monteagudo et al., 2017; Cadenas-Fernández et al., 2020; Wu et al., 2020). But the inoculation of inactivated ASFV vaccine cannot induce immune protection, even under the condition of new adjuvant compatibility (Arias et al., 2017; Cadenas-Fernández et al., 2021). The efficacy and safety of many gene-deleted ASFV vaccines have been improved. Nevertheless, the safety of the candidate strains remains to be fully evaluated (Tran et al., 2022; Ding et al., 2022a,b). The research strategies of ASFV subunit vaccine mainly include antigen-based vaccines, DNA vaccines, vector vaccines, and prime-boost immunization strategies. The main targets of these vaccines are ASFV structural proteins p30, p54, p72, pp62, and CD2v (Gaudreault and Richt, 2019). However, these antigens used cannot induce complete and lasting protection. This may be because these viral proteins can induce a kind of antibody that contribute to uptake of the virus into cells, resulting in antibody-dependent enhancement (ADE) of infection (Gaudreault and Richt, 2019). Previous studies have shown that high levels of antibodies lacking neutralizing activity may be detrimental and exacerbate disease progression (Blome et al., 2014; Sunwoo et al., 2019). Therefore, the screening and identification of ASFV antigens and epitopes with well-defined protective effects is essential for the development of subunit vaccines.

The p72 protein is encoded by the B646L gene and is the main structural protein of ASFV, accounting for 31 to 33% of the virus particle (Wang et al., 2019). p72 forms a homotrimer, with each monomer adopting a double jelly-roll structure that is comprised pseudo-hexameric capsomers (Liu et al., 2019). The protein is mainly involved in viral capsid assembly and is important in virus adsorption and invasion of susceptible cells (Duan et al., 2022). Besides, it induces an antibody response after viral infection. And due to its conservatism and immunogenicity, it is widely used as an antigen in the development of methods to diagnose (Caixia et al., 2022; Geng et al., 2022; Wan et al., 2022).

Detailed analyses of epitopes provide a deep understanding of the roles of amino acids in the epitopes and viral replication (Chen et al., 2021). However, few studies have addressed p72 epitopes. Eight linear B cell epitopes (Heimerman et al., 2018; Yin et al., 2022) and a single conformational neutralizing epitope (Borca et al., 1994) have been identified for p72.

In the present study, we obtained 17 monoclonal antibodies (mAbs) against p72, which did not show significant neutralizing activity. In addition, four novel and highly conserved linear epitopes of p72 were identified. We developed a competitive ELISA (cELISA) based on mAb-2B8D7. This cELISA method could specifically distinguish ASF-negative and positive sera. These results significantly increase the understanding of the epitopes of p72 and provide valuable information for the development of diagnostic methods and subunit vaccines.

## 2. Ethics statement

The animal experiments on mice were approved by the Animal Care and Use Committee of Lanzhou Veterinary Research Institute. The experimental procedures were performed in strict accordance with the recommendations in the nursing Guide, Laboratory Animals and Use, Ministry of Science and Technology, PRC.

## 3. Materials and methods

### 3.1. Experimental animals, viruses, cells, and serum samples

BALB/c, 6- to 8-week-old, female, specific pathogen-free mice were purchased from the Laboratory Animal Center of Lanzhou Veterinary Research Institute. The ASFV/II/CN/SC/2019 strain was preserved in the P3 biosafety laboratory of Lanzhou Veterinary Research Institute. SP2/0 and SF9 were stored in our laboratory. Commercially available primary porcine alveolar macrophages (PAMs) isolated from lung tissues of healthy pigs according to Carrascosa et al. (1982) were stored in liquid nitrogen. ASFV standard positive serum was purchased from China Institute of Veterinary Drug Control.

### 3.2. Expression and purification of p72 protein

The p72 gene was retrieved from the GenBank database (GenBank MH766894.1), optimized for synthesis, and cloned into a pET-SUMO vector. The recombinant plasmid was transferred into *Escherichia coli* BL21(DE3). A single colony that developed was incubated in LB medium at 37°C with shaking at 200 rpm until the optical density at 600 nm (OD600) was approximately 0.6. Then, isopropyl β-d-1-thiogalactopyranosid (IPTG) was added (final concentration 1 mM) to induce the expression of p72 protein during a 16 h incubation at 20°C. Bacteria were recovered by centrifugation at 5,000 g for 10 min. To purify p72, the bacteria were resuspended in inclusion body buffer (20 mM Tris, 300 mM NaCl, 10 mM EDTA, 1% Triton X-100, pH 8.0) and sonicated on ice. After centrifugation, the supernatant was removed, and the pelleted inclusion bodies were resuspended in IB solubilization buffer (20 mM Tris, 500 mM NaCl, 8 M urea, pH 8.0) and spun at 4°C overnight. Supernatants were harvested following centrifugation at 10,000 × *g* for 20 min. Each supernatant was purified using Ni-NTA and the eluted protein was analyzed for purity by SDS-PAGE. The purified protein was dialyzed for renaturation. In brief, the purified protein was placed in a dialysis bag (Spectrum Chemical, New Brunswick, NJ, United States) and renaturated in renaturation buffer 1 (4 M urea, 200 mM imidazole, 20 mM Tris, 250 mM Nacl buffer, 0.1% Triton-100, pH 8.0) for 12 h at 4°C. The dialysis bag was then transferred to renaturation buffer 2 (2 M urea, 100 mM imidazole, 20 mM Tris, 250 mM Nacl, 0.1% Triton-100, pH 8.0) for 12 h followed by renaturation buffer 3 (20 mM Tris, 250 mM Nacl, 0.1% Triton-100, 10% glycerol, 0.4 M arginine, 2 mM reduced glutathione, 0.2 mM oxidized glutathione, pH 8.0) for 12 h.

### 3.3. Preparation of p72 mAbs

The mice were immunized with 20 μg renaturated p72 protein emulsified in equal proportions with Freund’s complete adjuvant or Freund’s incomplete adjuvant (Sigma-Aldrich, St. Louis, MO, United States). The immunizations were performed four times with 2-week intervals. Three days after the fourth immunization, spleen cells were harvested and fused with SP2/0 cells. Confluent cells were cultured in Dulbecco’s modified Eagle’s medium (DMEM) containing HAT Supplement (Sigma-Aldrich) and 20% fetal bovine serum (FBS). After 8 days, supernatants from confluent cells were collected and detected by indirect ELISA using wells coated with p72 recombinant protein. Positive wells were selected and subcloned three times by limited dilution. The positive clones were cultured in DMEM containing 20% FBS and 1% penicillin–streptomycin.

To prepare ascites containing antibodies against the p72 protein, 0.5 ml of ascites adjuvant (BIODRAGON, Beijing, China) was injected into the abdominal cavity of 12-week-old female Balb/c mice. After 15 days, approximately 1.0 × 10^6^ hybridoma cells were injected into the abdominal cavity of the mice. Ascites was collected after 9–10 days and purified using affinity chromatography (HiTrap Protein G, GE, GA, United States). The purified antibodies were determined using indirect ELISA. Subtypes of mAbs were identified by SBA Clonotyping System-HRP Kit (Southern Biotech, Birmingham, AL, United States). Specificity of mAbs against recombinant proteins and viruses was evaluated by western blotting and immunofluorescence assay.

### 3.4. Indirect immunofluorescence assay

PAMs were seeded in 24-well plates (1.0 × 10^6^ cells/well; Corning, New York, NY, United States) cultured with RPMI-1640 (Gibco, Grand Island, NY, United States) containing 15% FBS. Twelve hours after seeding, PAMs were infected with ASFV/II/CN/SC/2019 at a multiplicity of infection of 0.1 for 72 h. The cells were washed three times with PBS and fixed with 4% paraformaldehyde for 1 h. After washing three times with PBS, the cells were permeabilized with 0.25% Triton X-100 for 10 min. After blocking with 5% BSA for 2 h, the cells were incubated with the hybridoma supernatant (1:10 dilution) at 37°C for 1 h. After washing with PBS, a 1:1000 dilution of tetramethyl rhodamine isothiocyanate (TRITC) conjugated goat anti-mouse IgG (Abcam, Cambridge, United Kingdom) was added and incubated for 1 h. After washing three times with PBS, the cells were observed by inverted fluorescence microscopy using a model DM16000B microscope (Leica Microsystems, Wetzlar, Germany).

### 3.5. Expression of p72 peptide fragments

**Figure 1:**
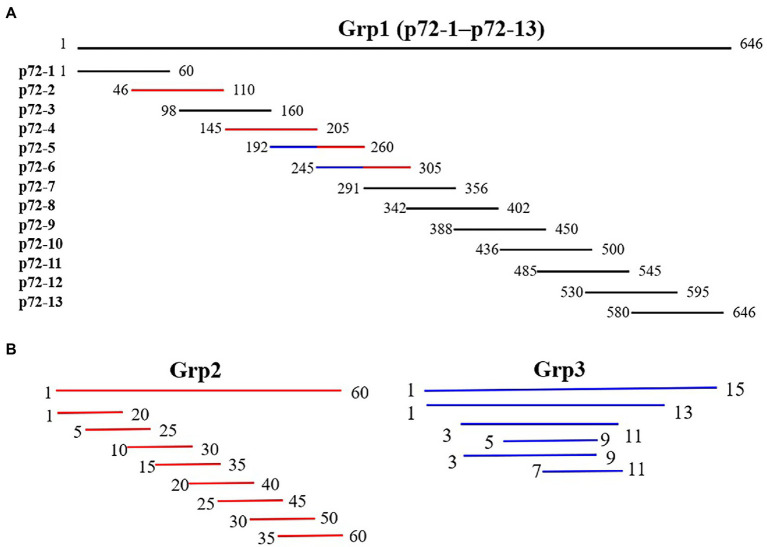
Diagram of overlapping fragments of p72. **(A)** Grp1 comprises 13 overlapping peptides (p72-1 to p72-13) based on full-length p72. **(B)** Grp2 (marked in red in Grp1) comprises the truncation mode of four peptides (p72-2, p72-4, p72-5, and p72-6). Grp3 (marked in the blue in Grp1) comprises the truncation mode of two overlapping peptides (p72-45, amino acids 192 to 205; p72-56, amino acids 245 to 260).

To locate the epitopes recognized by each mAb, a series of overlapping peptides were designed based on the full-length p72 gene (Figure 1). A total of 81 genes (S1–S81) listed in Supplementary Tables 1–3 were synthesized and cloned into the expression vector pGEX-4T-1. After being validated by DNA sequencing analysis, the constructed plasmid was transformed into *E. coli* BL21 (DE3), expressed as described above, and finally analyzed by SDS-PAGE and western blot analysis.

### 3.6. Sequence alignment

To verify the conservation of the seven epitopes in different ASFV genotypes, the representative sequences of 27 isolates from nine different genotypes were aligned using Clustal W to determine whether the mutation occurred in these epitopes.

### 3.7. SDS-PAGE and western blotting

Samples were mixed with 4× loading buffer and heated at 100°C for 10 min. Proteins in each sample were separated by 12% SDS-PAGE. After the gel was stained with Coomassie blue staining solution for 10 min, the bands were decolorized with decolorization solution and analyzed. For western blot analysis, protein samples were electro-transferred to polyvinylidene difluoride (PVDF) membranes (Bio-Rad, Hercules, CA, United States). After blocking with 5% skim milk powder at 37°C for 2 h, hybridoma cell supernatant dilution (1:10 dilution) was added and incubated overnight at 4°C. After washing three times with PBS-Tween (PBST), a 1:5000 dilution of horseradish peroxidase (HRP)-conjugated goat anti-mouse IgG (Abcam, Cambridge, United Kingdom) was added and incubated at 37°C for 1 h. After washing three times with PBST, enhanced chemiluminescence (ECL) reagent (Thermo Fisher Scientific, Waltham, MA, United States) was added. Blots were scanned and analyzed using the Odyssey CLx imaging system (LI-COR, Lincoln, NE, United States).

### 3.8. Indirect ELISA

Wells of 96-well plates were coated with p72 recombinant protein diluted in CBS to a final concentration of 0.25 μg/ml at 4°C overnight. Plates were washed three times and blocked with 5% skim milk at 37°C for 2 h. Hybridoma cell supernatant and ascites were diluted and added to the plate in a volume of 100 μl/well and incubated at 37°C for 30 min. After washing three times with PBST, goat anti-mouse IgG-HRP (1:10000 dilution) was added as the secondary antibody and incubated at 37°C for 30 min. After washing three times with PBST, 50 μl of 3,3′,5,5′-tetramethylbenzidine (TMB) substrate solution (Surmodics, Minneapolis, MN, United States) was added and incubated at 37°C for 10–15 min. Fifty microliter of 2 M H_2_S0_4_ was added, and OD values were measured at 450 nm using a microplate reader (Thermo Fisher Scientific).

### 3.9. Neutralization

PAMs adjusted to 1 × 10^6^/mL were added to wells of a 24-well culture plate and cultured in RPMI-1640 medium containing 15% FBS in an atmosphere of 5% CO_2_ at 37°C for 12 h. The purified mAbs, diluted to a final concentration of 5 μg/ml, were mixed with ASFV CN/SC/2019 (median tissue culture infectious dose [TCID_50_] of 200) and incubated at 4°C overnight. The mixture of virus and mAbs was inoculated into wells containing PAMs. After 1 h, the mixture solution was discarded. After washing three times with PBS, RPMI-1640 medium containing 5% FBS was added and cultured for 72 h. After three cycles of freezing and thawing in a culture plate, the ASFV genomes of each well were extracted using a virus genome extraction kit (AG, Hunan, China) and amplified by qPCR (Pro Taq HS Premix Probe kit; AG).

The inactivated ASFV CN/SC/2019 was used as a template for PCR amplification using primer p72-F (5′-GGAATTCCATATGGCATCAGGAGGAGC-3′) and p72-R (5′-CCGCTCGAGTTCTTAAACCCCGCAAAT-3′). PCR amplification and pET-28a vector were digested with BamH I and Xho I enzymes, then ligated with T4 ligase to form the recombinant plasmid pET28a-p72. The recombinant plasmid diluted 10 times continuously was used as template for qPCR amplification. The logarithmic value of plasmid concentration and Ct value were analyzed by linear regression. The copy number of the B646L gene in each sample was calculated according to the Ct value and the established standard curve. The virus neutralization rate (%) was calculated as:


$$\begin{array}{l} \mathrm{Neutralization}\;\left(\%\right)\\ =100\%-\frac{\mathrm{copy number of}\;B646L\;\mathrm{gene}\;\left(mAb-\mathrm{ASFV}\right)}{\mathrm{copy number of}\;B646L\;\mathrm{gene}\;\left(\mathrm{negative control}\right)}\% \end{array}$$

### 3.10. Competitive ELISA

To verify the diagnostic feasibility of the 17 mAbs, preliminary screening was performed by cELISA. In brief, wells of 96-well plates were coated with p72 recombinant protein diluted with CBS to a final concentration of 0.125–8 μg/ml at 4°C overnight. Wells were washed three times using PBST and then blocked with 5% skim milk at 37°C for 2 h. ASF standard positive and negative sera were diluted (1:5–1:640) and added to the wells (100 μl/well) and incubated at 37°C for 1 h. After washing three times with PBST, mAbs were diluted (1:1000–128,000) and added to the wells and incubated at 37°C for 30 min. After washing the wells three times with PBST, goat anti-mouse IgG-HRP (1:10000 dilution) was added and incubated at 37°C for 30 min. After washing three times with PBST, 50 μl of TMB substrate solution (Surmodics) was added and incubated at 37°C for 10–15 min. Fifty microliter of 50 μl 2 M H_2_SO_4_ was added, and OD values were measured at 450 nm using a microplate reader (Thermo Fisher Scientific). Using the determined optimized coating concentrations of antigens, dilutions of mAbs, and serum dilution, the ratios of negative serum to positive serum (N/P) were calculated. A greater N/P ratio indicated that ASFV-infected sera was better able to block the binding of mAbs to p72 protein.

## 4. Results

### 4.1. Production and identification of recombinant p72 protein

**Figure 2:**
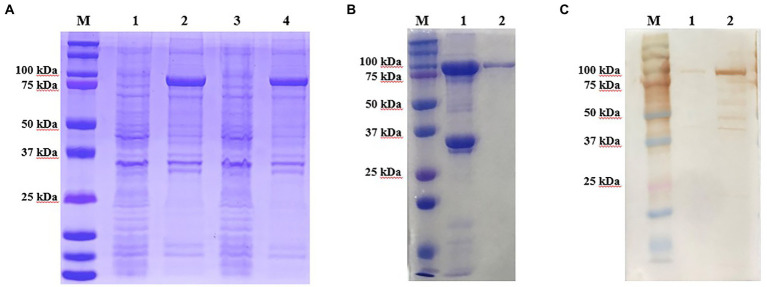
Production and identification of the p72 recombinant protein. **(A)** Expressed form of p72 recombinant protein in *E. coli*. M is the protein marker, lane 1 contains the bacterial lysate before induction, lane 2 contains the bacterial lysate after induction, lane 3 contains the supernatant after ultrasonic lysis of the bacteria, and lane 4 contains the precipitate after ultrasonic lysis of the bacteria. **(B)** Purification of the p72 recombinant protein. M is the protein marker, lane 1 contains the cell precipitate before purification, and lane 2 contains the purified p72 recombinant protein. **(C)** Reactivity of p72 recombinant protein with ASF-positive serum. M is the protein marker, lane 1 contains the pre-induction bacterial lysate, and lane 2 contains the post-induction bacterial lysate.

The recombinant p72 protein was induced and expressed in *E. coli*. SDS-PAGE confirmed that the expressed form of the protein was an inclusion body. As expected, the apparent molecular weight was approximately 100 kDa (p72 is approximately 72 kDa and the vector protein is approximately 18 kDa; Figure 2A). The expressed recombinant protein was purified by Ni-NTA; purity of the eluted protein was verified by SDS-PAGE (Figure 2B). The reactivity of the recombinant protein with ASF-positive serum was identified by western blotting (Figure 2C). After purification and renaturing, the p72 recombinant protein was immunized to generate 17 hybridoma cells capable of stably secreting p72-specific antibodies. Antibody isotype analysis identified 4F6D4, 4E10C9, 2B8B9, 2B8F7, 2B8D7, 2B7B6, 4E10B3, 6H2C5, 4E10B2, 4D4C8, 6D6D9, 5C9D6, and 2C9D6 as subclass IgG1; 5C9E3 and 3D4A9 as subclass IgG2a; 4F6B8 was as subclass IgG2b; and 6B10E8 as subclass IgM. All 17 mAbs possessed a kappa light chain.

### 4.2. Reactivity of the 17 mAbs

**Figure 3:**
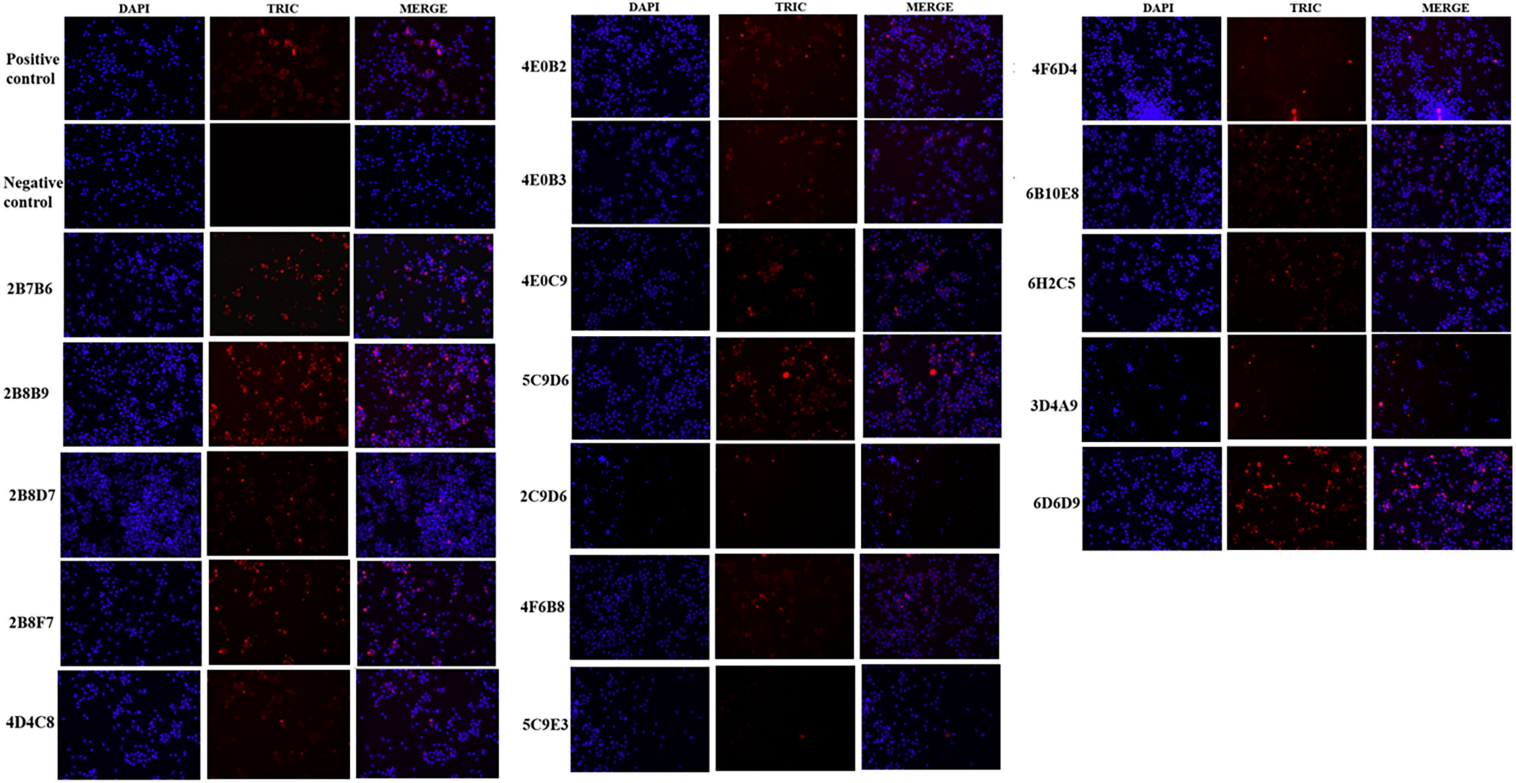
Reactivity of 17 mAbs in the indirect immunofluorescence assay (IFA). The positive control was ASF-positive serum and uninfected PAMs were used as the negative control.

**Figure 4:**
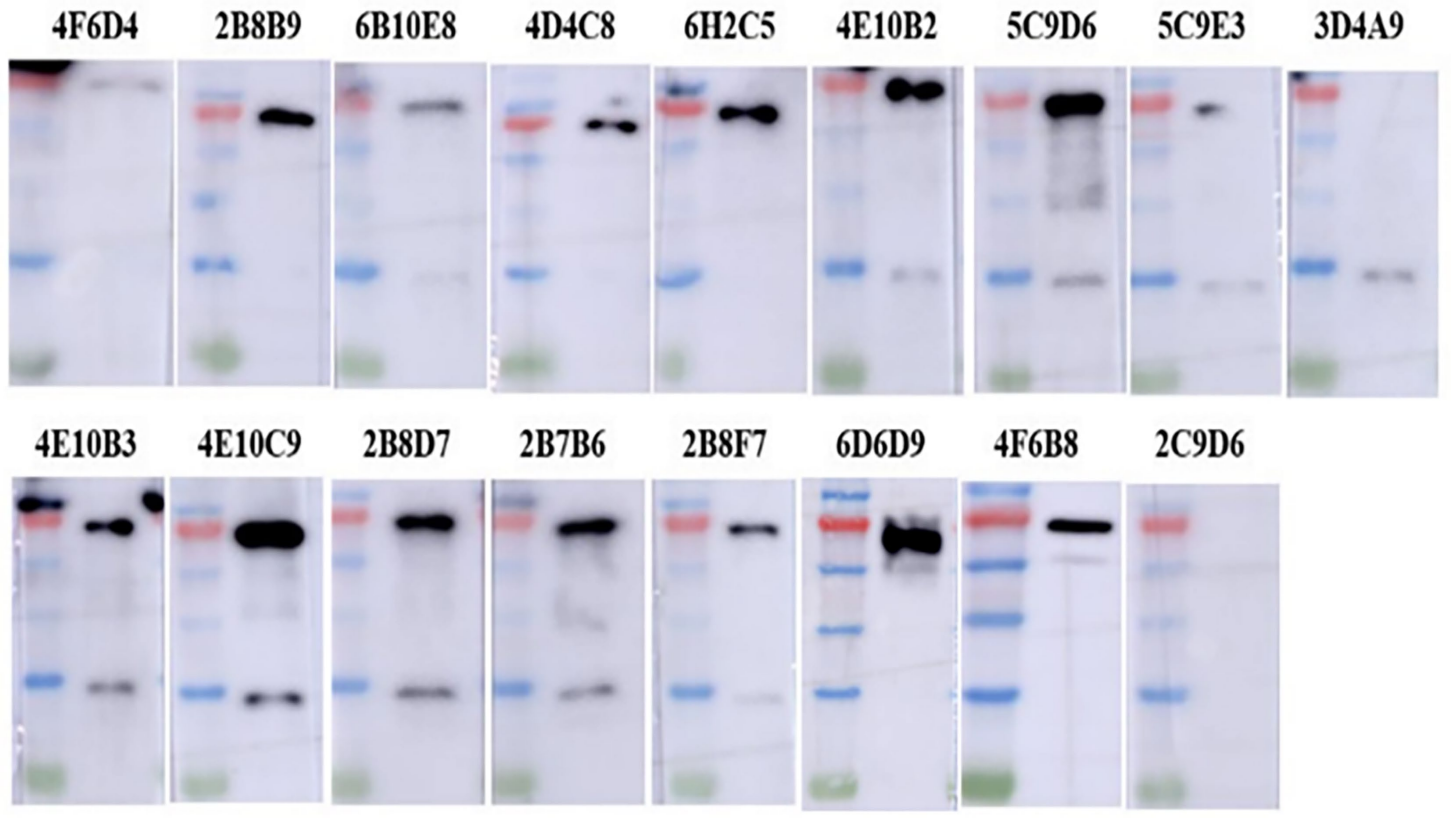
Reactivity of 17 mAbs against ASFV was preliminarily identified by WB. ASFV-infected PAMs were harvested at 72 h post-infection, electrophoresed on 12% SDS-PAGE gels and electro-transferred to polyvinylidene difluoride (PVDF) membranes, and detected with 17 mAbs as primary antibodies.

The IFA results indicated the reactivity of all 17 mAbs with ASFV. Thereinto, 3 mAbs (3D4A9, 5C9E3, and 2C9D6) had poor reactivity with ASFV (Figure 3). We further verified the reactivity of these 17 mAbs with ASFV-infected PAMs by WB. The results showed that the 4 mAbs (2B8B9, 5C9D6, 4E10C9, and 6D6D9) were highly reactive with ASFV, while the 3 mAbs (3D4A9, 5C9E3, and 2C9D6) were not reactive with ASFV (Figure 4).

### 4.3. Expression and identification of epitopes

**Figure 5:**
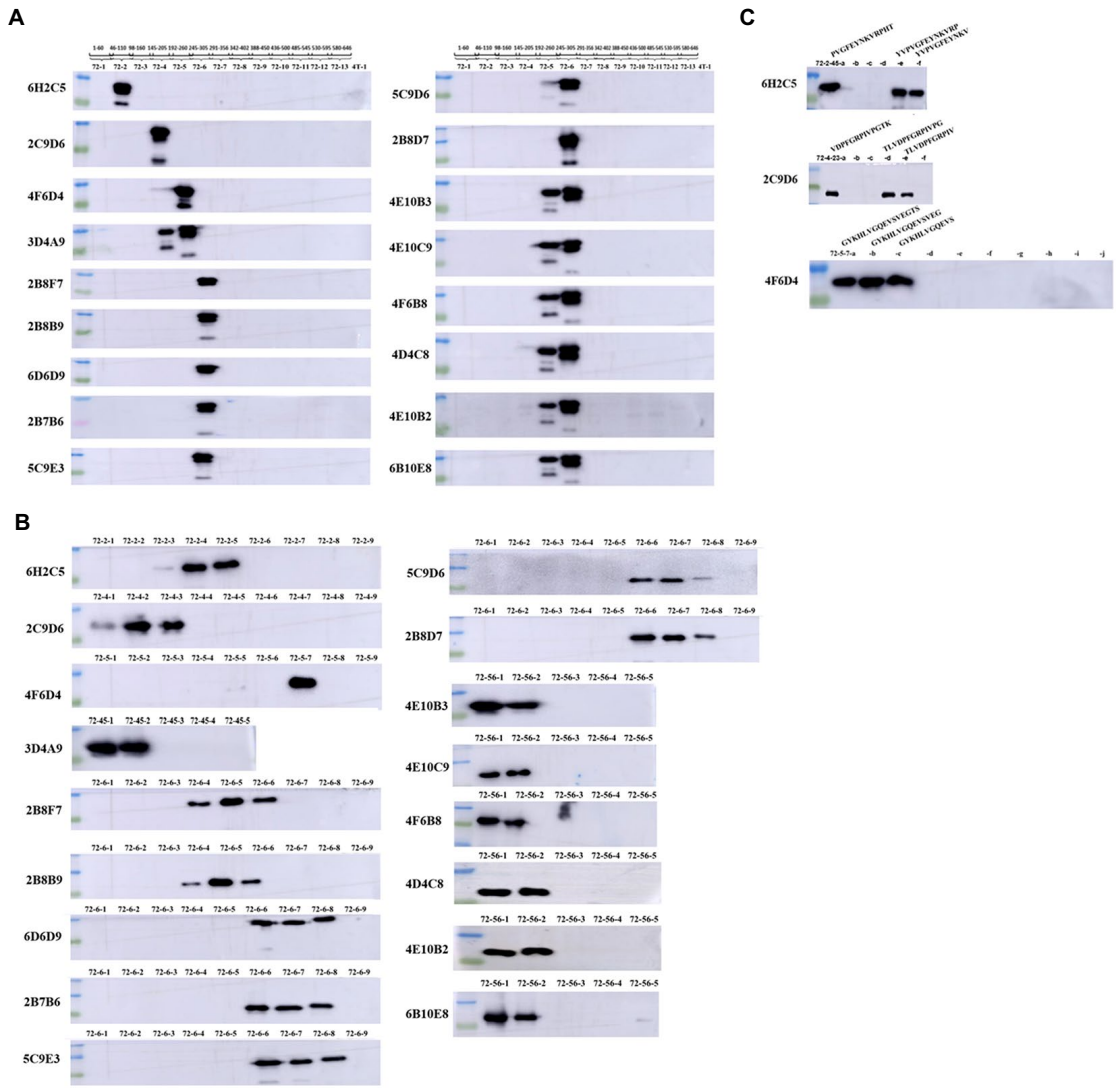
Chromatograms of 17 mAbs recognition epitopes. **(A)** The reactivity of 13 overlapping peptides (p72-1–p72-13) truncated based on full-length p72 with 17 mAbs. **(B)** The reactivity of truncated overlapping peptides based on six peptide fragments (p72-2, p72-4, p72-5, p72-45, p72-6 and p72-56, which have reactivities identified in **A**) with 17 mAbs. **(C)** To further define the epitopes identified by the 3 mAbs (6H2C5, 2C9D6, and 4F6D4), the N- or C-terminus of the peptide fragments (72–2-45, 72–4-23, 72–5-7) were sequentially subtracted by 2 or 3 amino acids and reacted with these 3 mAbs.

**Figure 6:**
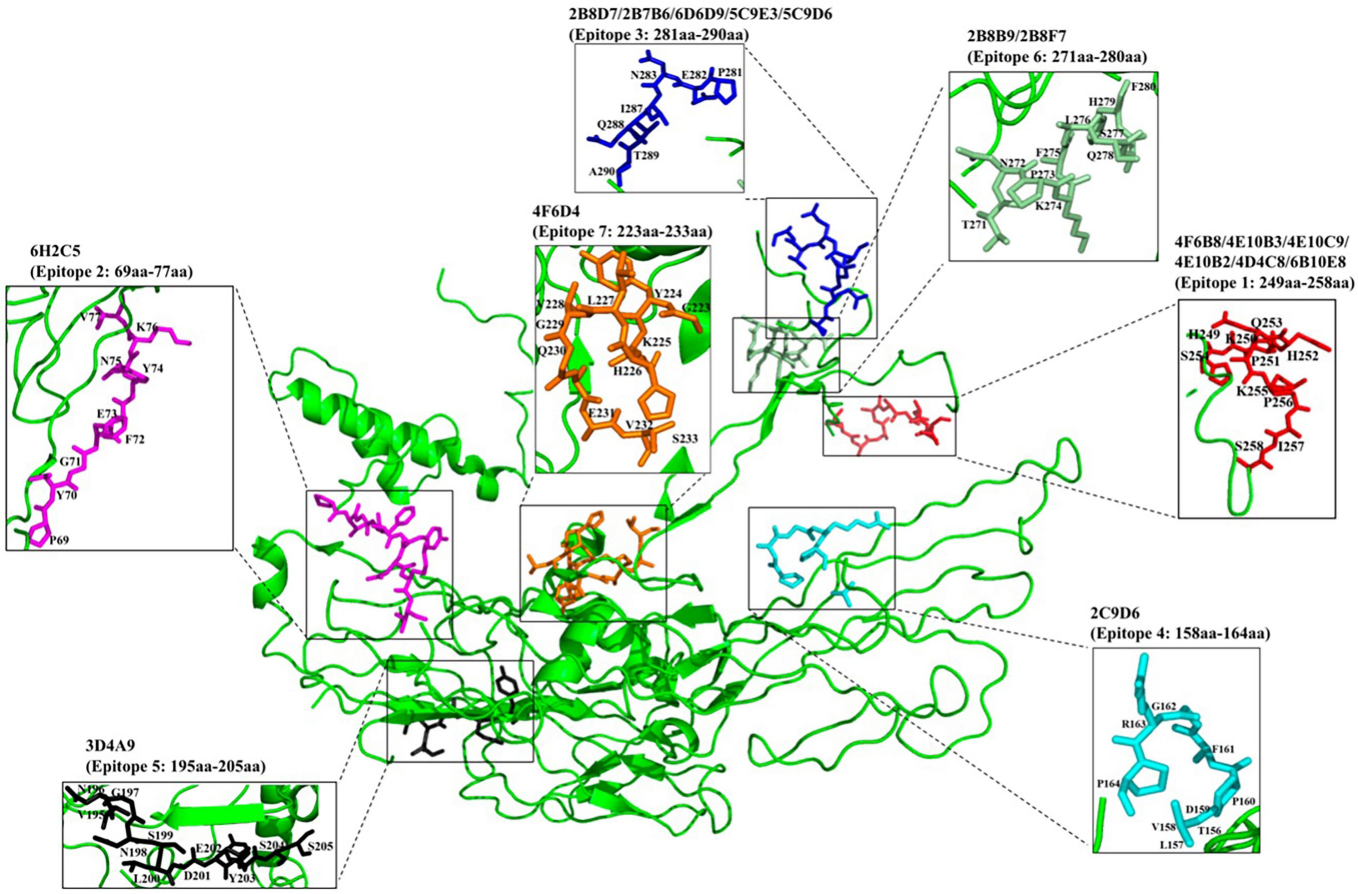
Localization of epitopes recognized by 17 mAbs in the structure of ASFV p72 protein.

To identify the epitopes recognized by the 17 mAbs, 13 overlapping peptide fragments (p72-1, p72-2, p72-3, p72-4, p72-5, p72-6, p72-7, p72-8, p72-9, p72-10, p72-11, p72-12, and p72-13) spanning p72 (1–646 amino acids) were expressed using an *E. coli* expression system and analyzed by western blotting. The recognized epitopes were located at p72-2 (6H2C5), p72-4 (2C9D6), p72-5 (4F6D4), p72-4 and − 72-5 (3D4A9), p72-6 (2B8F7, 2B8B9, 6D6D9, 2B7B6, 5C9E3, 5C9D6, and 2B8D7), and p72-5 and p72-6 (4E10B3, 4E10C9, 4F6B8, 4D4C8, 4E10B2, and 6B10E8; Figure 5A). To further determine the localization of these mAbs, we expressed overlapping peptides of p72-2 (cut into nine segments), p72-4 (cut into nine segments), p72-5 (cut into nine segments), p72-45 (cut into five segments), p72-6 (cut into nine segments), and p72-56 (cut into five segments). These were reacted with the mAbs. The recognized epitopes were ^195^VNGNSLDEYSS^205^ for 3D4A9; ^271^TNPKFLSQHF^280^ for 2B8F7 and 2B8B9; ^281^PENSHNIQTA^290^ for 6D6D9, 2B7B6, 5C9E3, 5C9D6, and 2B8D7; ^249^HKPHQSKPIL^258^ for 4E10B3, 4E10C9, 4F6B8, 4D4C8, 4E10B2, and 6B10E8. The identified amino acid sequences of the remaining three epitopes were longer. We further shortened them by successively subtracting two or three amino acids from the N- or C-terminus. Subsequent results showed that the recognized epitopes were ^69^PVGFEYNKV^77^ by 6H2C5, ^158^VDPFGRPIV^166^ by 2C9D6, and ^223^GYKHLVGQEVS^233^ by 4F6D4 (Figures 5B,C). The epitopes identified by these mAbs were mapped in the structure of ASFV p72 protein using PyMOL software (Figure 6).

### 4.4. Conservative analysis

**Figure 7:**
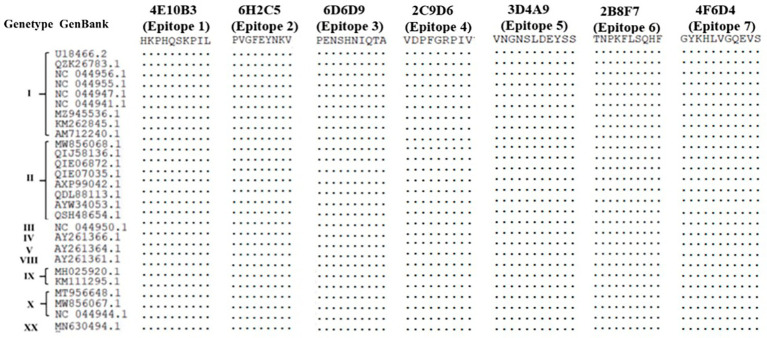
Sequence comparison of phenotypes identified using the 17 mAbs with different ASFV genotypic isolates.

Sequence alignment of 27 ASFV isolates from nine genotypes revealed that the seven epitopes identified by the mAbs obtained in this study were highly conserved (Figure 7).

### 4.5. Neutralization

**Figure 8:**
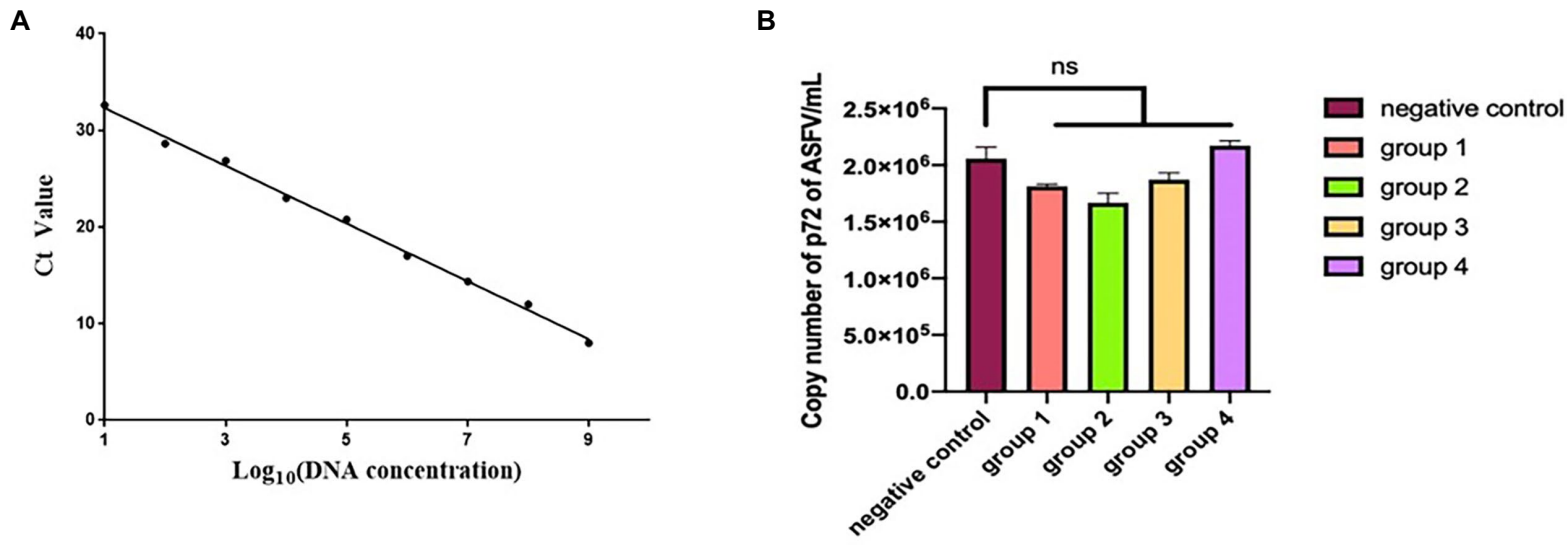
**(A)** Linear regression between the logarithmic value of plasmid concentration and Ct. **(B)** The number of copies of B646L gene was measured in group 1–4. Group 1 is the mixture of mAbs 6D6D9 (identify epitope 3) + 2B8F7 (identify epitope 6) and ASFV CN/SC/2019 (200 TCID_50_). Group 2 is the mixture of mAbs 4F6D4 (identify epitope 4) + 2C9D6 (identify epitope 7) and ASFV CN/SC/2019 (200 TCID_50_). Group 3 is the mixture of mAbs 4E10B3 (identify epitope 1) + 6H2C5 (identify epitope 2) and ASFV CN/SC/2019 (200 TCID_50_). Group 4 is the mixture of mAb 3D4A9 (identify epitope 5) and ASFV CN/SC/2019 (200 TCID_50_). Each virus-mAb mixture was incubated overnight at 4°C, inoculated into PAMs, and allowed to adsorb for 1 h. PAMs were cultured for 72 h and the ASFV genome was extracted and amplified by qPCR.

The standard curve drawn in Figure 8A is expressed as the linear equation: y = −2.9401x + 34.956 (R^2^ = 0.9923). According to the curve, seven mAbs recognizing seven epitopes (divided into four groups) were incubated with ASFV (200 TCID_50_) and inoculated into PAMs. The copy number of the B646L gene in each sample was calculated according to the Ct value and the established standard curve. Each group was repeated three times. The data were expressed as mean ± standard error of the mean, and the significance of the differences between groups was calculated by GraphPad Prism Software (San Diego, CA, United States). *p* > 0.05 indicated no significant difference (NS; Zhang et al., 2021). The copy number of the p72 gene did not differ significantly between the ASFV-mAbs and ASFV-negative control (*p* > 0.05). None of the anti-p72 mAbs displayed significant neutralizing activity against ASFV (Figure 8B).

### 4.6. Primary application of mAbs

**Figure 9:**
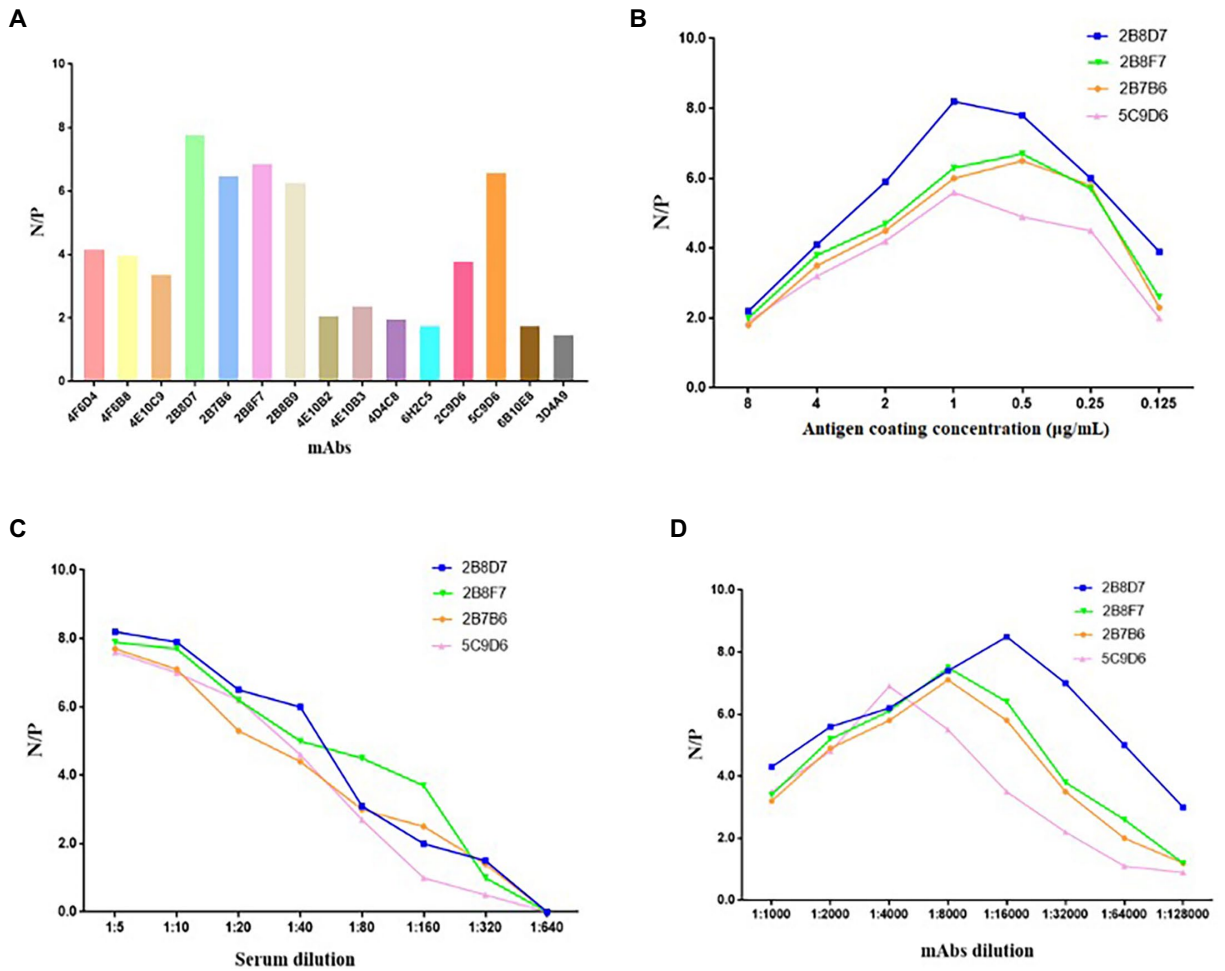
Screening of mAbs for ASFV diagnosis. **(A)** The 17 mAbs were preliminarily selected based on their N/P ratios in the same conditions of antigen coating concentration of 0.25 μg/ml, hybridoma cell supernatant dilution of 1: 5, and serum dilution of 1: 10. **(B)** N/P ratios of the four selected mAbs at different coating concentrations. **(C)** N/P ratios of the four mAbs at different dilutions of sera in the condition of optimum coating concentrations (1 μg/ml of 2B8D7 and 2B8F7, 0.5 μg/ml of 2B7B6 and 5C9D6). **(D)** N/P ratios of the four mAbs at different dilutions in the condition of optimum coating concentrations of antigens (1 μg/ml of 2B8D7 and 2B8F7, 0.5 μg/ml of 2B7B6 and 5C9D6) and optimum serum dilution (1:5).

To determine whether the 17 mAbs could be used in diagnosis, these mAbs were preliminarily selected according to their N/P ratio in the same conditions of an antigen coating concentration of 0.25 μg/ml, hybridoma cell supernatant dilution of 1:5, and serum dilution of 1:10 (Figure 9A). Only four mAbs (2B8D7, 2B8F7, 2B7B6, and 5C9D6) displayed an N/P ratio > 7. We further optimized the conditions of the four mAbs to obtain the maximum N/P ratio. The optimal coating antigen concentration, serum dilution, and optimal dilution of the four mAbs concentration were screened and determined in a stepwise manner (Figures 9B–D). The N/P ratio of mAb 2B8D7 was the highest. Therefore, mAb-2B8D7 was chosen to verify the feasibility in the diagnosis. Using the optimum conditions, six positive sera and one negative serum with known background were detected. Each sample was calculated using the formula: percentage inhibition (PI) = [1– (tested sample OD_450_/standard negative OD_450_)] × 100%. The results showed that the PI values of six positive sera were 93.56, 95.13, 92.61, 91.25, 90.15, and 88.63%, and PI values of negative serum were 9.34%. Therefore, the preliminary results show that mAb-2B8D7 could distinguish negative and positive sera.

## 5. Discussion

ASFV has a history of more than 100 years worldwide (Gaudreault et al., 2020). Yet, currently, there is still no commercial vaccine. Improving the level of biosafety is still the main means to prevent and control the ASF (Danzetta et al., 2020). p72 displays good reactivity and immunogenicity. Hence, it is one of the most commonly detected antigens in infected pigs (Liu et al., 2019; Li et al., 2022) and is used in subunit vaccine preparations (Lokhandwala et al., 2016; Lopera-Madrid et al., 2017; Cadenas-Fernández et al., 2020). Identifying epitopes of p72 protein will contribute to the development of subunit vaccines and serological diagnostics. In addition, mapping the p72 viral protein epitope and defining the degree of conservation of the identified epitope could also enhance the current understanding of the antigenic structure and virus-antibody interaction.

To obtain more comprehensive mAbs and expand the epitope map of p72, the full-length p72 protein was presently expressed using prokaryotic system to immunize the mice. Seven linear B cell epitopes (epitopes 1–7) were identified using the 17 mAbs obtained. Epitope 4 (^158^VDPFGRPIV^166^) recognized by mAb 2C9D6, epitope 6 (^271^TNPKFLSQHF^280^) recognized by mAbs 2B8F7 and 2B8B9, and epitope 3 (^281^PENSHNIQTA^290^) recognized by mAbs 6D6D9, 2B7B6, 5C9E3, 5C9D6, and 2B8D7 were similar to the study by Heimerman et al. (^156^TLVDPFGRPI^165^, ^265^QRTCSHTNPKFLSQHF^280^, and ^280^FPENSHNIQTAGKQD^294^; Heimerman et al., 2018). Epitope 2 (^69^PVGFEYENKV^77^) recognized by mAb 6H2C5, epitope 7 (^223^GYKHLVGQEV^233^) recognized by mAb 4F6D4, epitope 5 (^195^VNGNSLDEYSS^205^) recognized by mAb 3D4A9, and epitope 1 (^249^HKPHQSKPIL^258^) recognized by mAbs 4E10B3, 4E10C9, 4F6B8, 4D4C8, 4E10B2, and 6B10E8 have never been reported in previous studies. Furthermore, four linear epitopes (^31^SNIKNVNKSY^40^, ^41^GKPDP^45^, ^56^HLVHFNAH^63^, and ^185^ERLYE^189^) have been reported by Yin et al. (2022).

The neutralization test performed to evaluate the neutralization potency of these seven classes of mAbs did not show significant neutralization activity. Furthermore, four mAbs that recognize four linear epitopes also have no neutralizing ability in the study by Yin et al. (2022). But, in the study of Borca et al. (1994), mAb 135D4 that is identified to recognize the conformational epitope on p72 has partially neutralized activity. Besides, exposed region 1 (ER1) and ER2 from the crown of the p72 capsomer orient toward the outside of the capsid, which may contribute to form a conformational epitope (Wang F. et al., 2021). The β-strands of ER3 and ER4 within the same subunit constitute a four-stranded β-sheet that forms the head of the p72 capsomer. This may be another conformational epitope. And these four ERs probably define neutralizing epitopes (Wang F. et al., 2021). These results indicate that the neutralized epitopes may be conformational epitopes. Seven epitopes identified in our study were linear epitopes, probably because the p72 protein that we used to immunize mice was a non-natural refolded structural protein. In conclusion, it highlights the need for further studies to identify the p72 conformational epitope.

The epitopes recognized by the 17 mAbs are highly conserved in different genotypes of ASFV. Accordingly, we preliminarily evaluated the application of these mAbs in diagnosis. The results showed that mAb-2B8D7 had the highest N/P ratio and could specifically distinguish positive and negative serum, indicating the potential of this mAb in diagnostic methods. The mAb-2B8D7 could be further used to develop the magnetic particle chemiluminescence immunoassay to realize the automated, high-throughput, and rapid detection in future (Zhang and Qi, 2011; Feng et al., 2014; Fu et al., 2018).

In conclusion, we expressed and purified full-length p72 protein through prokaryotic expression systems. The protein was used as the antigen to immunize mice to prepare 17 specific mAbs. These mAbs had no neutralizing ability. Seven linear B cell epitopes were identified (epitopes 1–7). Four of the linear epitopes (epitopes 1, 2, 5, and 7) are novel. These epitopes are highly conserved among the nine genotypes of ASFV. In addition, mAb-2B8D7 that recognizes epitope 3 (Heimerman et al., 2018) was screened and its potential value in cELISA was indicated. The collective findings may provide a solid foundation for further studies of p72 epitope and ASF diagnosis by magnetic particle chemiluminescence immunoassay.

## Data availability statement

The datasets presented in this study can be found in online repositories. The names of the repository/repositories and accession number(s) can be found in the article/Supplementary material.

## Ethics statement

The animal study was reviewed and approved by Animal Ethics Committee of LVRI, CAAS.

## Author contributions

HC and JS performed the conceptualization and funding acquisition. WL performed the formal analysis, review and editing, and funding acquisition. CM and SY performed the methodology, formal analysis, clinical sample detection methodology, and writing-original draft. GZ, YM, SW, ZH, and DP performed the material preparation investigation. All authors contributed to the article and approved the submitted version.

## Funding

This study was financially supported by the National Natural Science Foundation of China (32273038) and the National Key Research and Development Program of China (2021YFD11801402).

## Conflict of interest

The authors declare that the research was conducted in the absence of any commercial or financial relationships that could be construed as a potential conflict of interest.

## Publisher’s note

All claims expressed in this article are solely those of the authors and do not necessarily represent those of their affiliated organizations, or those of the publisher, the editors and the reviewers. Any product that may be evaluated in this article, or claim that may be made by its manufacturer, is not guaranteed or endorsed by the publisher.

## Supplementary material

The Supplementary material for this article can be found online at: https://www.frontiersin.org/articles/10.3389/fmicb.2023.1126794/full#supplementary-material
