# Supplementary material for: Identification of p72 epitopes of African swine fever virus and preliminary application
Source: Front Microbiol. 2023 Feb 3;14:1126794. doi: 10.3389/fmicb.2023.1126794 (PMC9935695; doi:10.3389/fmicb.2023.1126794)
Supplement: Supplementary file 1 [file Table_1.DOCX]

Supplementary Table 1

Supplementary Table 1 comprises 13 overlapping peptides (p72-1 to p72-13) based on full-length p72.

| Polypeptide fragment | | amino acid sequences (5’ - 3’) |
| --- | --- | --- |
| p72 (1-13) | S1 | MASGGAFCLIANDGKADKIILAQDLLNSRISNIKNVNKSYGKPDPEPTLSQIEETHLVHF |
|  | S2 | PTLSQIEETHLVHFNAHFKPYVPVGFEYNKVRPHTGTPTLGNKLTFGIPQYGDFFHDMVGHHIL |
|  | S3 | DFFHDMVGHHILGACHSSWQDAPIQGTSQMGAHGQLQTFPRNGYDWDNQTPLEGAVYTLVDP |
|  | S4 | NQTPLEGAVYTLVDPFGRPIVPGTKNAYRNLVYYCEYPGERLYENVRFDVNGNSLDEYSS |
|  | S5 | FDVNGNSLDEYSSDVTTLVRKFCIPGDKMTGYKHLVGQEVSVEGTSGPLLCNIHDLHKPHQSKPILTD |
|  | S6 | HDLHKPHQSKPILTDENDTQRTCSHTNPKFLSQHFPENSHNIQTAGKQDITPITDATYLD |
|  | S7 | KQDITPITDATYLDIRRNVHYSCNGPQTPKYYQPPLALWIKLRFWFNENVNLAIPSVSIPFGERF |
|  | S8 | LAIPSVSIPFGERFITIKLASQKDLVNEFPGLFVRQSRFIAGRPSRRNIRFKPWFIPGVI |
|  | S9 | RNIRFKPWFIPGVINEISLTNNELYINNLFVTPEIHNLFVKRVRFSLIRVHKTQVTHTNNNH |
|  | S10 | RVHKTQVTHTNNNHHDEKLMSALKWPIEYMFIGLKPTWNISDQNPHQHRDWHKFGHVVNAIMQP |
|  | S11 | DWHKFGHVVNAIMQPTHHAEISFQDRDTALPDACSSISDISPVTYPITLPIIKNISVTAH |
|  | S12 | PITLPIIKNISVTAHGINLIDKFPSKFCSSYIPFHYGGNAIKTPDDPGAMMITFALKPREEYQPS |
|  | S13 | ITFALKPREEYQPSGHINVSRAREFYISWDTDYVGSITTADLVVSASAINFLLLQNGSAVLRYST |

Supplementary Table 2

Supplementary Table 2 comprises the truncation mode of 6 peptides (p72-2, p72-4, p72-5, p72-6, p72-45 and p72-56).

| Polypeptide fragment | | amino acid sequences (5’ - 3’) |
| --- | --- | --- |
| p72-2 (1-9) | S14 | PTLSQIEETHLVHFNAHFKP |
|  | S15 | IEETHLVHFNAHFKPYVPVG |
|  | S16 | LVHFNAHFKPYVPVGFEYNK |
|  | S17 | AHFKPYVPVGFEYNKVRPHT |
|  | S18 | YVPVGFEYNKVRPHTGTPTL |
|  | S19 | FEYNKVRPHTGTPTLGNKLT |
|  | S20 | VRPHTGTPTLGNKLTFGIPQ |
|  | S21 | GTPTLGNKLTFGIPQYGDFF |
|  | S22 | GNKLTFGIPQYGDFFHDMVGHHIL |
| p72-4 (1-9) | S23 | NQTPLEGAVYTLVDPFGRPI |
|  | S24 | EGAVYTLVDPFGRPIVPGTK |
|  | S25 | TLVDPFGRPIVPGTKNAYRN |
|  | S26 | FGRPIVPGTKNAYRNLVYYC |
|  | S27 | VPGTKNAYRNLVYYCEYPGE |
|  | S28 | NAYRNLVYYCEYPGERLYEN |
|  | S29 | LVYYCEYPGERLYENVRFDV |
|  | S30 | EYPGERLYENVRFDVNGNSL |
|  | S31 | RLYENVRFDVNGNSLDEYSS |
| p72-5 (1-9) | S32 | FDVNGNSLDEYSSDVTTLVR |
|  | S33 | NSLDEYSSDVTTLVRKFCIP |
|  | S34 | YSSDVTTLVRKFCIPGDKMT |
|  | S35 | TTLVRKFCIPGDKMTGYKHL |
|  | S36 | KFCIPGDKMTGYKHLVGQEV |
|  | S37 | GDKMTGYKHLVGQEVSVEGT |
|  | S38 | GYKHLVGQEVSVEGTSGPLL |
|  | S39 | VGQEVSVEGTSGPLLCNIHD |
|  | S40 | SVEGTSGPLLCNIHDLHKPHQSKPILTD |
| p72-6 (1-9) | S41 | HDLHKPHQSK PILTDENDTQ |
|  | S42 | PHQSKPILTDENDTQRTCSH |
|  | S43 | PILTDENDTQ RTCSHTNPKF |
|  | S44 | ENDTQRTCSHTNPKFLSQHF |
|  | S45 | RTCSHTNPKFLSQHFPENSH |
|  | S46 | TNPKFLSQHFPENSHNIQTA |
|  | S47 | LSQHFPENSHNIQTAGKQDI |
|  | S48 | PENSHNIQTAGKQDITPITD |
|  | S49 | NIQTAGKQDITPITDATYLD |
| p72-45 (1-5) | S50 | FDVNGNSLDEY |
|  | S51 | VNGNSLDEYSS |
|  | S52 | GNSLDEYSS |
|  | S53 | VNGNSLDEY |
|  | S54 | SLDEY |
| p72-56 (1-5) | S55 | HDLHKPHQSKPIL |
|  | S56 | LHKPHQSKPILTD |
|  | S57 | PHQSKPIL |
|  | S58 | LHKPHQSKP |
|  | S59 | HQSKP |

Supplementary Table 3

Supplementary Table 3 comprises the truncation mode of 3 peptides (p72-2-45, p72-4-23 and p72-5-7).

| Polypeptide fragment | | amino acid sequences (5’ - 3’) |
| --- | --- | --- |
| p72-2-45 (1-6) | S60 | PVGFEYNKVRPHT |
|  | S61 | GFEYNKVRPHT |
|  | S62 | EYNKVRPHT |
|  | S63 | NKVRPHT |
|  | S64 | YVPVGFEYNKVRP |
|  | S65 | YVPVGFEYNKV |
| p72-4-23 (1-6) | S66 | VDPFGRPIVPGTK |
|  | S67 | PFGRPIVPGTK |
|  | S68 | GRPIVPGTK |
|  | S69 | TLVDPFGRPIVPG |
|  | S70 | TLVDPFGRPIV |
|  | S71 | TLVDPFGRP |
| p72-5-7 (1-10) | S72 | GYKHLVGQEVSVEGTSG |
|  | S73 | GYKHLVGQEVSVEG |
|  | S74 | GYKHLVGQEVS |
|  | S75 | GYKHLVGQ |
|  | S76 | GYKHL |
|  | S77 | HLVGQEVSVEGTSGPLL |
|  | S78 | GQEVSVEGTSGPLL |
|  | S79 | VSVEGTSGPLL |
|  | S80 | EGTSGPLL |
|  | S81 | SGPLL |
